# Burden of Invasive Group B *Streptococcus* Disease and Early Neurological Sequelae in South African Infants

**DOI:** 10.1371/journal.pone.0123014

**Published:** 2015-04-07

**Authors:** Ziyaad Dangor, Sanjay G. Lala, Clare L. Cutland, Anthonet Koen, Lisa Jose, Firdose Nakwa, Tanusha Ramdin, Joy Fredericks, Jeannette Wadula, Shabir A. Madhi

**Affiliations:** 1 Medical Research Council: Respiratory and Meningeal Pathogens Research Unit, Faculty of Health Sciences, University of the Witwatersrand, Johannesburg, Gauteng, South Africa; 2 Department of Science and Technology/National Research Foundation: Vaccine Preventable Diseases, Faculty of Health Sciences, University of the Witwatersrand, Johannesburg, Gauteng, South Africa; 3 Department of Paediatrics, Faculty of Health Sciences, University of the Witwatersrand, Johannesburg, Gauteng, South Africa; 4 Department of Clinical Microbiology and Infectious Diseases, National Health Laboratory Service, University of the Witwatersrand, Johannesburg, Gauteng, South Africa; 5 National Institute for Communicable Diseases: a division of National Health Laboratory Service, Centre for Vaccines and Immunology, Sandringham, Gauteng, South Africa; Faculdade de Medicina de Lisboa, PORTUGAL

## Abstract

**Introduction:**

Group B *Streptococcus* (GBS) is a leading cause of neonatal sepsis and meningitis. We aimed to evaluate the burden of invasive early-onset (0–6 days of life, EOD) and late-onset (7–89 days, LOD) GBS disease and subsequent neurological sequelae in infants from a setting with a high prevalence (29.5%) of HIV among pregnant women.

**Methods:**

A case-control study was undertaken at three secondary-tertiary care public hospitals in Johannesburg. Invasive cases in infants <3 months age were identified by surveillance of laboratories from November 2012 to February 2014. Neurodevelopmental screening was done in surviving cases and controls at 3 and 6 months of age.

**Results:**

We identified 122 cases of invasive GBS disease over a 12 month period. Although the incidence (per 1,000 live births) of EOD was similar between HIV-exposed and HIV-unexposed infants (1.13 vs. 1.46; p = 0.487), there was a 4.67-fold (95%CI: 2.24–9.74) greater risk for LOD in HIV-exposed infants (2.27 vs. 0.49; p<0.001). Overall, serotypes Ia, Ib and III constituted 75.8% and 92.5% of EOD and LOD, respectively. Risk factors for EOD included offensive draining liquor (adjusted Odds Ratio: 27.37; 95%CI: 1.94–386.50) and maternal GBS bacteriuria (aOR: 8.41; 95%CI: 1.44–49.15), which was also a risk-factor for LOD (aOR: 3.49; 95%CI: 1.17–10.40). The overall case fatality rate among cases was 18.0%. The adjusted odds for neurological sequelae at 6 months age was 13.18-fold (95%CI: 1.44–120.95) greater in cases (13.2%) than controls (0.4%).

**Discussion:**

The high burden of invasive GBS disease in South Africa, which is also associated with high case fatality rates and significant neurological sequelae among survivors, is partly due to the heightened risk for LOD in infants born to HIV-infected women. An effective trivalent GBS conjugate vaccine targeted at pregnant women could prevent invasive GBS disease in this setting.

## Introduction

There has been slow progress in the decline of neonatal mortality rates in developing countries where severe bacterial infections accounted for an estimated 680 000 neonatal deaths in 2012 [1, 2]. Group B *streptococcus* (GBS) has been recognized as a leading contributor of neonatal sepsis and meningitis in developed countries, even though intra-partum antibiotic prophylaxis (IAP) is routinely administered to GBS-colonized pregnant women [3–5]. Additionally, neurodevelopmental problems are seen in about 22–50% of infants surviving GBS meningitis [6–10]. In developing countries, such as South Africa, where GBS screening and IAP during pregnancy is not standard-of-care, the mortality rate from invasive GBS disease is higher than in developed countries (10–60% compared to 7–11%) [11–13]. Furthermore, in South Africa, the high prevalence of maternal HIV-infection (29.5%) [14] is likely to aggravate the burden of invasive GBS disease [15]. We therefore prospectively determined the incidence of invasive GBS disease, including the effect of maternal HIV-infection on disease burden in infants born in Johannesburg. Furthermore, we evaluated risk factors for invasive GBS disease and assessed early neurodevelopmental sequelae in GBS-affected infants and healthy controls.

## Methods

Between November 2012 and February 2014, we undertook a case-control study at the three largest academic hospitals in Johannesburg; namely Chris Hani Baragwanath Academic Hospital (CHBAH), Charlotte Maxeke Johannesburg Academic Hospital and Rahima Moosa Mother and Child Hospital. The standard-of-antenatal care for the prevention of invasive GBS in neonates does not include universal screening for recto-vaginal GBS colonization during pregnancy although IAP is provided to women who have risk factors such as maternal fever and prolonged rupture of membranes (≥18 hours prior to delivery). Blood and cerebrospinal fluid (CSF) cultures are routinely performed in infants admitted with suspected sepsis or meningitis. HIV infection testing is routinely performed in pregnant women and confirmed using two independent rapid antibody screening tests [16]. Pregnant women with a CD4+ lymphocyte count >350 cells/mm^3^ and WHO stage 1 and 2 received antiretroviral prophylaxis with zidovudine (AZT); whilst those with CD4+ lymphocyte count ≤350 cells/mm^3^ or WHO stage 3 or 4 were initiated on triple antiretroviral therapy (ART). From April 2013, all pregnant women irrespective of CD4+ lymphocyte count were initiated on ART [16, 17].

Invasive GBS disease (cases) were defined as an infant <90 days of age in whom GBS was cultured from blood, CSF or other normally sterile sites; or when GBS was identified in CSF by latex agglutination. Cases were identified by ZD through daily surveillance of the pediatric wards and microbiology services at the three hospitals. Early-onset disease (EOD) was defined when GBS was isolated in infants younger than seven days of life, and infants between 7–89 days of age with GBS disease were regarded as having late-onset disease (LOD).

Control subjects were matched for: (i) gestational age to term, or within 2 weeks for cases born <37 weeks gestation, (ii) maternal HIV-infection status, (iii) maternal age (within 2.5 years of the case mother’s age), and (iv) enrollment within 0–6 days after birth for EOD cases and within 14 days (but >7 days of life) of chronological age for LOD cases. Controls for EOD were selected from admission and labor wards at CHBAH, whereas controls for LOD were identified through the birth registries and contacted telephonically for possible study-enrolment. For cases born at ≥34 weeks gestational age, at least 5 controls (mean: 7; range: 5–14) were matched for EOD and 3 controls (mean: 5; range: 3–7) for LOD. For cases born at <34 weeks gestational age, at least one control (mean: 2; range: 1–5) was matched for EOD and at least one control (mean: 2, range: 1–4) for LOD. All controls were clinically well at enrolment, and followed up to confirm they did not develop invasive GBS disease.

Cases and controls were followed up at 3 and 6 months of the infant’s chronological age. These visits were carried out by either one of three trained research assistants or by ZD. At these visits, the infant’s underwent neurological and development examinations and were screened using the Denver Developmental Screening Test II (Denver-II). The Denver-II makes a valuable screening tool (83% sensitivity) with a high degree of test-retest and inter-examiner reliability [18, 19]. The Denver-II tests 4 domains; gross-motor, fine-motor, language and personal-social. Each test item is represented horizontally as a percentile age range (25–90%) for which it is normally estimated that the item can be achieved. A “fail” or “refusal” by the infant in an item to the left of the age line is classified as a “delay”, whilst a “fail” or “refusal” by the infant in an item through the 75–90% age percentile is classified as a “caution”. The final result was then scored as “normal” (no delays or 1 caution) or “suspect/abnormal” (≥2 cautions or ≥1 delay) in each of the four domains. We defined neurological sequelae as an abnormal Denver-II developmental screening test for any of the four domains or hypertonia and/or hyper-reflexia detected on examination. Infants with developmental delay were referred to occupational, physical and/or speech therapists. Visual and hearing assessments were not routinely tested on participants.

### Laboratory methods

GBS was isolated from blood samples using the Bact/Alert microbial system (Organon Teknika, Durham, NC). Positive specimens were subsequently plated on blood or chocolate agar incubated both aerobically and at 35 degrees under 5–10% CO2, and observed for colony growth for 72 hours. Gram-staining was performed on CSF samples, which were also plated onto blood or chocolate agar plates, inoculated into an enrichment broth (Brain Heart Infusion, Diagnostics Media Production) and observed for colony growth for 72 hours. Specimens were also analyzed by a GBS antigen agglutination test if the CSF cell counts were suggestive of bacterial meningitis. Positive GBS isolates were serotyped and stored.

Although screening for maternal GBS colonization is not a routine investigation in Johannesburg, maternal colonization status was determined for participants enrolled in the study by separately swabbing the lower vagina and rectum using Rayon tipped swabs and charcoal-free Amies transport medium (Medical Wire Equipment Co. Ltd. Cat: MW170). In addition, a mid-stream urine specimen was also cultured. Mothers of cases and controls were swabbed at the time of enrolment, while controls matched to EOD were swabbed immediately after delivery. Swabs were plated onto CHROMAgar StrepB plates (Media Mage Cat: M10155) which were incubated at 37°C for 18–24 hours in aerobic conditions and examined for growth of mauve GBS-like colony morphologies. Identified colonies were subjected to further confirmatory tests, such as the catalase test, growth on bile esculin agar, inability to hydrolyze esculin, Christie Atkinson Munch-Petersen (CAMP) test and B antigen latex agglutination test [20]. Serotyping for GBS types Ia, Ib, II to IX was performed using latex agglutination (Statens Serum Institute, SSI, Sweden) [21]. Non-typeable and discordant isolates were further characterized by a single-plex PCR method for serotypes Ia, Ib, II, III, IV and V using primer sequences described by Poyart et al. [22].

### Statistical analysis

The incidence (per 1,000 live births) of invasive GBS disease over a twelve month period was calculated as the number of cases (EOD or LOD) in black-African infants that specifically resided in regions D and G of the Johannesburg metropolitan area. We only included black African infants with GBS disease residing in these specified regions because the care-givers of these infants predominantly access health care at either CHBAH or RMMCH. We did not undertake incidence calculation for non-black African infants or black-African infants not residing in regions D and G because these infants were likely to utilize other health care facilities not under surveillance in the study. There were 31504 live births over 12 months in regions D and G; 8827 (28%) infants were born to HIV-infected women [23].

For proportions, Chi-square or Fischer’s exact test were used to compare demographic and clinical characteristics between cases of EOD and LOD. Medians were reported for non-parametric variables and compared using the Wilcoxon rank-sum (Mann-Whitney) test. Serotype distributions were reported as proportions of the total number of cases serotyped and stratified by EOD and LOD.

Univariate analysis was used to identify risk factors for invasive GBS disease, predictors of infant mortality and to compare neurological sequelae. For the multivariate analysis, adjusted odds ratios (aOR) using conditional logistic regression was used to adjust for variables with p-values <0.15 detected by univariate analysis. For the identification of risk factors predisposing to invasive GBS disease, we also included gestational age, maternal age and HIV status. For neurological sequelae, we adjusted for factors that may impact on neurodevelopment; including, gender, gestational age, birth weight <2500 grams, perinatal asphyxia, mechanical ventilation, infant HIV-exposure status and previous non-GBS-related hospitalizations. Data was analyzed using STATA version 13.1 (College Station, Texas, USA). Two-tailed p-values <0.05 were considered statistically significant. The study was approved by the University of Witwatersrand Human Research Ethics Committee (HREC number: M120963). Written informed consent was obtained from mothers of infants at enrolment for participation in the study.

## Results

There were 122 infants (<90 days-of-age) with invasive GBS disease over a 12 month period, including 82 (67.2%) at CHBAH, 22 (18.0%) at CMJAH and 18 (14.8%) at RMMCH. Most infants (n = 116; 95.1%) were of black-African descent and 48 (39.4%) of all infants were born to HIV-infected mothers. Sixty six (54.1%) infants had EOD, of which 63 (95.5%) were identified within the first 24 hours of life. The predominant clinical presentation was sepsis (97.0%) and meningitis (58.9%) in infants with EOD and LOD, respectively (Table 1).Overall, 44 (36.1%) cases occurred in infants born before 37 completed gestational weeks; EOD occurred significantly more commonly than LOD in prematurely-born infants (45.4% versus 25.0%; p = 0.019; Table 1). Recurrence of invasive GBS disease occurred in two infants (1.6%), and one case and one control were diagnosed as HIV-infected at 6 weeks of age. Group B Streptococcus was cultured in 119 (97.5%) cases, whilst 3 (2.5%) cases of meningitis were identified on GBS latex agglutination of CSF samples, Of the 35 cases of meningitis, 6 (17.1%) had >250 red cells/μl in their CSF.

**Table 1:** Demographic characteristics of infants with invasive Group B *Streptococcal* (GBS) disease.

	All cases, n = 122	EOD^1^, n = 66	LOD^2^, n = 56	OR(95%CI)^3^	p-value^4^
Gestational Age					
≥37 weeks	78 (63.9)	36 (54.6)	42 (75.0)	0.40 (0.17–0.93)	0.019
<37 - ≥34 weeks	14 (11.5)	8 (12.1)	6 (10.7)	1.15 (0.32–4.31)	0.808
<34 weeks	30 (24.6)	22 (33.3)	8 (14.3)	3.00 (1.13–8.56)	0.015
Birth Weight					
≥2500 grams	77 (63.1)	38 (57.6)	39 (69.6)	0.59 (0.26–1.33)	0.169
1500–2499 grams	27 (22.1)	14 (21.2)	13 (23.2)	0.89 (0.35–2.30)	0.791
1000–1499 grams	10 (8.2)	7 (10.6)	3 (5.4)	2.10 (0.45–13.12)	0.292
≤999 grams	8 (6.6)	7 (10.6)	1 (1.8)	6.53 (0.79–299.28)	0.068
Gender					
Male	68 (55.7)	35 (53.0)	33 (58.9)	0.79 (0.36–1.72)	0.513
Race					
Black	116 (95.1)	62 (93.9)	54 (96.4)	0.57 (0.05–4.20)	0.526
Mixed race	6 (4.9)	4 (6.1)	2 (3.6)		
Maternal HIV status					
HIV-infected	48 (39.4)	17 (25.8)	31 (55.4)	0.27 (0.12–0.64)	<0.001
HIV-uninfected	73 (59.8)	48 (72.7)	25 (44.6)	2.67 (1.15–6.24)	0.012
HIV-unknown	1 (0.8)	1 (1.5)			
Mode of delivery					
Caesarean-section	29 (23.8)	20 (30.3)	9 (16.1)	2.27 (0.87–6.25)	0.066
Vertex delivery	91 (74.6)	45 (68.2)	46 (82.1)	0.47 (0.18–1.18)	0.078
Unknown	2 (1.6)	1 (1.5)	1 (1.8)		
GBS isolation					
Blood only	87 (71.3)	64 (97.0)	23 (41.1)	45.91 (10.04–410.36)	<0.001
CSF^5^ only	13 (10.7)		13 (23.2)		<0.001
Blood and CSF	22 (18.0)	2 (3.0)	20 (35.7)	0.06 (0.01–0.26)	<0.001
Infant age at presentation					
Median(range)	0 (0–74)	0 (0–5)	15 (7–74)		
<24hours	63 (51.6)	63 (95.5)			
1–6 days	3 (2.5)	3 (4.5)			
7–28 days	41 (33.6)		41 (73.2)		
>28 days	15 (12.3)		15 (26.8)		

^1^EOD-Early-onset disease.

^2^LOD-Late-onset disease.

^3^OR(95%CI)-calculated odds ratio with 95% confidence comparing EOD to LOD.

^4^p-value-using Chi-squared, Fischer exact or Wilcoxon rank-sum (Mann-Whitney) test.

^5^CSF-Cerebrospinal fluid.

HIV-exposed infants were 3.50 (95% CI: 1.53–8.09) times more likely to suffer from LOD than EOD. Additionally HIV-exposed infants were 6.85 (95% CI: 2.64–18.31) fold more likely to have GBS meningitis than HIV-unexposed infants. The CSF biochemistry and cytology parameters were similar between HIV-exposed and-unexposed infants: median CSF protein (p = 0.203), glucose (p = 0.364), polys (p = 0.984) and lymphs (p = 0.813).

### Incidence and serotype distribution of invasive GBS disease

Of 31 504 live births, there were 75 cases of invasive GBS disease in black-African infants residing in regions D and G; 73 (89.0%) infants presented to CHBAH and 2 (11.1%) to RMMCH. The overall incidence (per 1,000 live births) of invasive GBS disease was 2.38 (95% CI: 1.87–2.98); the incidences of EOD (n = 43) and LOD (n = 32) were 1.37 (95% CI: 0.99–1.84) and 1.02 (95%CI: 0.70–1.43) respectively. The estimated incidence of disease was significantly higher in HIV-exposed than in HIV-unexposed infants [3.40 (95% CI: 2.29–4.85) versus 1.94 (95% CI: 1.41–2.60) respectively; p = 0.016]. The incidence of EOD was similar in HIV-exposed (1.13; 95%CI: 0.54–2.08) and HIV-unexposed (1.46; 95%CI: 1.00–2.04; p = 0.487) infants but the incidence risk ratio of LOD was 4.67 (95% CI: 2.24–9.74) greater in HIV-exposed (2.27; 95% CI: 1.39–3.50) compared to HIV-unexposed infants (0.49; 95%CI: 0.24–0.87; p<0.001). Among the 66 cases of EOD; 32 (48.5%) were caused by serotype Ia, 5 (7.6%) by serotype Ib, 3 (4.5%) by serotype II, 13 (19.7%) by serotype III, 1 (1.5%) by serotype IV and 12 (18.2%) by serotype V. Among the 56 cases of LOD; 15 (26.8%) were caused by serotype Ia, 34 (60.7%) by serotype III, 4 (7.1%) by serotype V and 3 (5.4%) were not typed. Serotype III was the commonest (n = 23; 71.9%) cause of GBS meningitis, followed by serotype Ia (n = 8; 25.0%)

### Risk factors for early-onset GBS invasive disease

Offensive draining liquor (aOR: 27.37; 95% CI: 1.94–386.50) was a risk factor for EOD, whereas maternal GBS bacteriuria was a risk factor for EOD (aOR: 8.41; 95% CI: 1.44–49.15) and LOD (aOR: 3.49; 95% CI: 1.17–10.40) (Table 2). Maternal fever (≥38°C) was observed in only one case. Although the occurrence prolonged (>18 hours prior to delivery) rupture of membranes (PROM) was more common in EOD cases than controls, no increased risk was found in the multivariate analysis (p = 0.213) (Table 2). Thirteen (12.8%) cases mothers were not swabbed at enrollment. The prevalence of GBS colonization was higher in EOD cases (74.5%) than controls (25.1%). Maternal risk factors were not different in HIV-infected and -uninfected mothers (S1 Table).

**Table 2:** Risk factors for invasive Group B *Streptococcal* (GBS) disease in early-onset and late-onset disease cases and matched controls.

	Cases	Controls	Univariate-OR (95%CI)^1^	p-value	Multivariate-OR (95%CI)^2^	p-value
**Early-onset disease**	n = 56	n = 323				
Maternal GBS colonization	35/47 (74.5)	81/323 (25.1)	8.71 (4.15–19.23)	<0.001	3.38 (0.77–14.83)	0.107
Prolonged ROM (>18hours)^3^	14/49 (28.6)	32/313 (10.2)	3.51 (1.57–7.54)	<0.001	2.08 (0.61–7.08)	0.239
Maternal fever (≥38.0°C)^4^	1/50 (2.0)	0/319 (0)		0.136		
Offensive liquor	10/52 (19.2)	1/317 (0.3)	75.24 (10.05–3274.04)	<0.001	27.37 (1.94–386.50)	0.014
Maternal GBS Bacteriuria	27/47 (57.5)	22/220 (10.0)	12.15 (5.51–26.79)	<0.001	8.41 (1.44–49.15)	0.018
Any IAP^5^	7/31 (22.6)	40/104 (38.5)	0.47 (0.16–1.26)	0.103		
IAP ≥4 hours prior to delivery	5/31 (16.1)	36/104 (34.6)	0.36 (0.10–1.08)	0.074		
No IAP	24/31 (77.4)	64/104 (61.5)	2.14 (0.80–6.41)	0.103		
**Late-onset disease**	n = 46	n = 212				
Maternal GBS colonization	28/42 (66.7)	64/212 (30.2)	4.63 (2.17–10.11)	<0.001	2.44 (0.88–6.79)	0.088
Prolonged ROM(>18hours)^3^	2/35 (5.7)	18/204 (8.8)	0.63 (0.07–2.83)	0.746		
Offensive liquor	2/38 (5.3)	3/203 (1.5)	3.70 (0.30–33.27)	0.178		
Maternal GBS Bacteriuria	18/42 (42.9)	25/212 (11.8)	5.61 (2.48–12.46)	<0.001	3.49 (1.17–10.40)	0.025
Any IAP	1/12 (8.3)	16/56 (28.6)	0.23 (0.01–1.84)	0.269		
IAP ≥4 hours prior to delivery	1/12 (8.3)	10/56 (17.9)	0.42 (0.01–3.59)	0.674		
No IAP	11/12 (91.7)	40/56 (71.4)	4.40 (0.54–201.01)	0.269		

^1^Univariate-OR(95%CI)-calculated odds ratio with 95% confidence using Fischer exact test comparing cases and controls.

^2^ Multivariate-OR(95%CI)-calculated odds ratio with 95% confidence of disease using conditional logistic regression (For early-onset disease: adjusted for HIV-status, maternal age at delivery, gestational age, maternal GBS colonization, prolonged ROM, offensive liquor, maternal temperature>38, GBS bacteriuria and any intra-partum antibiotics. For late-onset disease: adjusted for HIV-status, maternal age at delivery, gestational age, maternal GBS colonization and GBS bacteriuria).

^3^ Prolonged ROM (>18 hours)-prolonged rupture of membranes.

^4^Maternal fever during labor.

^5^IAP-Intrapartum antibiotic prophylaxis to pregnant women that met risk-based criteria (gestation <37 weeks, PROM and maternal intra-partum fever).

Intra-partum antibiotic prophylaxis (IAP) was not administered to most mothers who had at least one risk factor (per Center for Disease Control risk based criteria for IAP; i.e. gestation <37 weeks, PROM and maternal intra-partum fever) predisposing to neonatal GBS disease [24]. Among EOD cases, 5 (16.1%) of 31 mothers with at least one risk factor received IAP ≥4 hours prior to delivery, two (6.5%) received IAP within 4 hours of delivery and 24 (77.4%) did not receive IAP during labor. Among controls, 36 (34.6%) of 104 mothers with at least one risk factor received IAP ≥4 hours prior to delivery, four (3.9%) received IAP within 4 hours of delivery and 64 (61.5%) did not receive IAP during labor. For infants born to mothers who received IAP at least 4 hours before delivery, the odds of acquiring EOD was 0.36 (95% CI: 0.10–1.08).

### Clinical presentation of GBS invasive disease

Infants with EOD presented most frequently with respiratory distress (83.3%), whilst other clinical and laboratory signs of sepsis occurred less frequently (<15%) (S2 Table). Respiratory distress was less common among LOD (35.7%) than EOD cases (p<0.001), but pyrexia occurred more frequently in LOD (39.3% vs 3.0%; p<0.001). As compared to EOD, infants with LOD also had an increased odds of presenting with poor feeding (OR: 20.71; 95% CI: 4.54–187.69), irritability (OR: 16.65; 95% CI: 5.03–69.74) and lethargy (OR: 3.37; 95% CI 1.17–10.51), and were more likely to have CRP >40 mg/l (58.7% vs 30.5%; p = 0.004) and leucopenia (37.5% vs 12.5%; p = 0.001) (S2 Table)

### Mortality and neurological outcomes of GBS invasive disease

The overall case fatality rate among cases was 18.0% (22/122), including 22.7% (15/66) for EOD and 12.5% (7/56) for LOD. Most deaths (14/22; 63.6%) occurred within 48 hours of hospital admission or birth. Twenty three (18.9%) infants were admitted to intensive care, of whom 19 (10 EOD and 9 LOD) required mechanical ventilation and 8 (5 EOD and 3 LOD) required inotropic support (Table 3). The mortality rate among infants requiring ventilation was 60.0% (n = 6) for EOD and 55.6% (n = 5) for LOD, and seven (87.5%) infants requiring inotropic support demised. Significant infant predictors of mortality were gestational age <34 weeks (aOR: 9.45; 95% CI: 2.11–42.29), apnea at presentation (aOR: 16.54; 95% CI: 1.55–176.33), seizures (aOR: 6.71; 95% CI: 1.07–42.24) or the need for inotropic support (aOR: 281.93; 95% CI: 7.32–10864.64) (Table 3). HIV-exposed infants were not at increased risk of death (aOR: 0.14; 95% CI: 0.02–0.79).

**Table 3:** Predictors of mortality from invasive Group B streptococcus (GBS) disease.

	Demised, n = 22	Survived, n = 100	Univariate-OR (95%CI)^1^	p-value	Multivariate-OR (95%CI)^2^	p-value
Timing of disease						
Early-onset disease	15 (68.2)	51 (51.0)	2.06 (0.71–6.47)	0.143	1.31 (0.29–5.95)	0.726
Late-onset disease	7 (31.8)	49 (49.0)	0.49 (0.16–1.41)	0.143		
Mode of presentation						
Meningitis	5 (22.7)	30 (30.0)	0.69 (0.18–2.18)	0.608		
Gestational age						
<34 weeks	11 (50.0)	19 (19.0)	4.26 (1.42–12.58)	0.002	9.45 (2.11–42.29)	0.003
HIV-exposure						
HIV-exposed	4 (18.2)	44 (44.0)	0.28 (0.07–0.95)	0.030	0.14 (0.02–0.79)	0.027
HIV-unexposed	17 (77.3)	56 (56.0)	2.67 (0.85–9.92)	0.092		
HIV-unknown	1 (4.5)					
Gender						
Male	11 (50.0)	57 (57.0)	0.75 (0.27–2.12)	0.549		
Clinical features						
Apnea	7 (31.8)	6 (6.0)	7.31 (1.79–29.7)	<0.001	16.54 (1.55–176.33)	0.020
Seizures	5 (22.7)	8 (8.0)	3.38 (0.76–13.34)	0.058	6.71 (1.07–42.24)	0.043
High/intensive care						
Mechanical Ventilation support	11 (50.0)	8 (8.0)	11.5 (3.31–40.06)	<0.001	0.34 (0.03–3.77)	0.376
Inotropic support	7 (31.8)	1 (1.0)	46.2 (5.09–2101.36)	<0.001	281.93 (7.32–10864.64)	0.002
Lab markers						
WCC^3^(<5x10^9^/l)	6 (27.3)	23 (23.0)	1.26 (0.36–3.88)	0.670		
CRP^4^(>40mg/l)	6 (27.3)	39 (39.0)	0.59 (0.17–1.76)	0.302		

^1^OR(95%CI)-calculated odds ratio with 95% confidence comparing infants that demised versus survivors of GBS disease using Chi-squared or Fischer exact test.

^2^ Multivariate-OR(95%CI)-calculated odds ratio with 95% confidence using logistic regression (adjusted for timing of disease, HIV-exposure, prematurity (<34 weeks), ventilation, inotropic support, apnea, seizures).

^3^WCC-White cell count.

^4^CRP-C-reactive protein.

Of the 100 surviving cases discharged from hospital, both the three and six monthly follow-ups were completed for 63 cases and 214 controls; whilst a further 10 cases and 66 controls only attended one of the two visits (S3 Table). Reasons for follow-up data being unavailable in the remaining cases included 6 whose parents declined for study participation, 4 cases born to women considered unable to provide informed consent and 17 cases were lost to follow-up. At 3 months of age, there were concerns about normal neurological development in 9 of 68 (13.2%) infants with invasive GBS disease and 1 of 262 (0.4%) control infants (Table 4). GBS-affected infants were 21.48 (95% CI: 2.58–179.15; p = 0.005) times more likely have neurological sequelae than controls. Three cases; one with hypertonia and one with an personal-social delay on Denver-II subsequently showed signs of recovery from neurological impairment at 6 months, whilst one case did not attend the visit.

**Table 4:** Neurological sequelae of infants with invasive Group B *Streptococcus* (GBS) disease at 3 and 6 month visits.

	Cases			Controls	Univariate-OR (95%CI) ^1^	p-value	Multivariate-OR (95%CI) ^2^	p-value
	Sepsis	Meningitis	Overall					
**3 months**	**n = 49**	**n = 19**	**n = 68**	**n = 262**				
Overall^3^	3 (6.1)	6 (31.6)	9 (13.2)	1 (0.4)	39.81 (5.27–1751.09)	<0.001	21.48 (2.58–179.15)	0.005
Abnormal Denver-II assessment^4^	2 (4.1)	1 (5.3)	3 (4.4)	1 (0.4)				
Hypertonia/hyper-reflexia^5^	1 (2.0)	5 (26.3)	6 (8.9)	0				
**6 months**	**n = 51**	**n = 17**	**n = 68**	**n = 232**				
Overall	5 (9.8)	4 (23.5)	9 (13.2)	1 (0.4)	35.24 (4.66–1550.57)	<0.001	13.18 (1.44–120.95)	0.023
Abnormal Denver-II assessment	4 (7.8)	1 (5.9)	5 (7.4)	1 (0.4)				
Hypertonia/hyper-reflexia	1 (2.0)	3 (17.6)	4 (5.9)	0				

^1^ Univariate-OR(95%CI)- calculated Odds ratio with 95% confidence using Fischer exact test comparing overall cases and controls

^2^ Multivariate-OR(95%CI)- calculated Odds ratio with 95% confidence using conditional logistic regression (adjusted for gender. gestational age, birth weight ≥2500, perinatal asphyxia, ventilation at presentation, HIV-status and previous non-GBS admissions).

^3^Number (%) of cases and controls with neurological sequelae based on abnormal Denver-II assessments and hypertonia/hyper-reflexia.

^4^Abnormal Denver-II assessments in four tested domains (Gross Motor, Fine Motor, Language and Personal/Social).

^5^Hypertonia and/or hyper-reflexia on neurological examination of infant with a normal Denver-II assessment.

At 6 months of age, four additional cases had an abnormal Denver-II screening test. Amongst the cases; two had fine-motor delay only, one had gross-motor delay only, one had gross and fine-motor delay and one had gross, fine-motor and personal-social delay. Four cases had hypertonia and/or hyper-reflexia on neurological examination with a normal Denver-II assessment. The only control with an abnormal Denver-II screening test had gross motor delay. GBS-affected infants were 13.18 (95% CI: 1.44-120.95; p = 0.023) times more likely have neurological sequelae than controls. Neurological abnormalities were detected in a greater proportion of GBS-affected infants with meningitis (23.5%) than sepsis (9.8%). Hydrocephalus was confirmed in two infants with meningitis.

## Discussion

Our study confirms the high incidence of invasive GBS disease (2.38 per 1 000 live births) observed in the last two decades in South Africa [11, 25], which is about twice the overall incidence in Africa (1.21; 95%CI: 0.50–1.91) and other regions [13]. Furthermore, we observed a five-fold greater risk of LOD in HIV-exposed compared to HIV-unexposed infants. The observed case fatality rate (18.0%) was similar to that previously reported [11]; this rate is lower than rates reported for Kenya (46%) and Malawi (33%) but almost double the rates reported in high income settings (7–11%) [12, 13]. Concerns about neurological development were noted in a significant proportion (13.2%) of infants with invasive GBS disease surviving to 6 months-of-age.

Unlike the declining trend of EOD in the United States (USA), most likely due to the implementation of IAP [26], there has been no significant change in the incidence rates of EOD in South Africa [11]. The lack of recognition of risk-factors for invasive GBS disease by staff, the late presentation of expectant mothers to antenatal facilities, and the severely under-staffed delivery units are likely factors to explain why only a quarter of women eligible for IAP received this therapy timeously even though the majority of births (±99%) occur in health-care facilities.

Maternal GBS bacteriuria, which is a surrogate marker of heavy recto-vaginal colonization, was significantly associated with EOD and LOD. In our study, maternal GBS bacteriuria was identified in 43% of mothers of LOD cases, of which almost 90% were infected with the same serotype that was isolated from maternal urine sample. These finding strongly support that IAP should be provided to mothers with GBS bacteriuria as it may be a risk factor for both EOD and LOD [24].

In keeping with the higher morbidity caused by infectious diseases in HIV-exposed infants in low-middle income countries [27, 28], the high maternal HIV prevalence (29.5%) may account, in part, for the high burden of invasive GBS disease in South Africa. Although the incidence of LOD among HIV-unexposed infants in our setting is similar to that seen in the USA and other countries [5, 13], we found that HIV-exposed infants were at a greater risk of developing LOD compared to their unexposed peers, as reported [15]. The reasons for this are unclear but may be related to perturbations of the infant immune system caused by exposure to HIV virion *in-utero* or maternal ART [29]; or lower levels of transferred maternal antibody predisposing HIV-exposed infants to invasive GBS disease [30]. Notably, no significant difference was observed when comparing CD4+counts amongst mothers of cases of LOD and controls (data not shown).

Significant predictors for invasive GBS disease related-death in our study included premature birth, apnea and/or seizures; which are indicators of severe illness in neonates [31]. Contrary to previous reports, in our study, HIV-exposure did not predict mortality in infants with invasive GBS disease [28]. Most deaths (63.6%) occurred within 48 hours of hospitalization, highlighting the fulminant nature of invasive GBS disease. Neurological sequelae was noted in a higher proportion of infants surviving GBS meningitis, similar to other reports [6]. The relatively low overall risk of neurological sequalae in our setting may also in part be related to the high mortality in these infants. Furthermore, in the absence of screening for auditory and visual deficits, as well as the early assessments, we are likely to have underestimated the number of infants with neurological sequelae from invasive GBS disease. There have been previous reports of long-term neurological sequelae in 26–50% of GBS meningitis survivors at 3–18 years of age [7–10], and we are continuing follow-up of children in this study to evaluate their long-term neurological outcomes.

Our results show that serotype Ia, instead of serotype III, is now the commonest (48.5%) cause of EOD in South Africa [11, 32]. In keeping with results from high-income countries [33, 34], the proportion of EOD and LOD caused by serotype V is increasing in South Africa [35]. Although there are differences in the invasive potential of different GBS serotypes, with serotype III being most invasive [32], temporal changes in serotype distribution associated with recto-vaginal colonization are mirrored by changes in their relative contribution to EOD as observed with serotype Ia over a twenty-year surveillance period in the United Kingdom [36]. Molecular characterization has however recognized the highly invasive ST-17 clone to be associated with serotype III invasive disease [37]. Nevertheless, the majority of serotypes causing EOD (76%) and LOD (93%) in our study were due to serotypes Ia, Ib and III, which are included in a trivalent polysaccharide protein conjugate vaccine targeted at immunization of pregnant women currently in clinical trials [38]

Limitations of our study include case enrolments over a single year; nevertheless, we identified a large number of invasive GBS cases and report a persistently high incidence of invasive GBS disease. Due to study constraints, we did not blind examiners performing neurodevelopmental screening tests but plan to do so at future visits. Although other developmental screening test are available (i.e. Bailey), we were limited to using the Denver-II screening test which has been shown to be reliable in young infants [19]. Furthermore, we currently only report on neurological sequelae up to 6 months of age, and did not have any follow-up outcomes on 27% of cases discharged from hospital. The short-term follow-up for neurological sequelae could fail to identify mild development delay or learning problems that manifest later in life, or conversely may over-estimate the long-term sequelae as the neurological system matures in children [39]. We were also unable to identify any significant differences in neurodevelopmental outcomes in HIV-exposed and-unexposed infants due to a small sample of infants with neurological sequelae.

Maternal vaccination effectively protects young infants against diseases such as tetanus, influenza and pertussis until 6 months of age [40–42]. Our study emphasizes the need to consider targeted vaccination of pregnant women for the prevention of invasive GBS disease in low-resource settings with a high prevalence of maternal HIV infection and where screening for recto-vaginal GBS colonization and IAP administration is not logistically feasible. An experimental trivalent GBS vaccine has been reported poorly immunogenic in HIV-infected pregnant women [43] and the immunogenicity of newer GBS conjugate vaccines therefore needs to be urgently evaluated in settings with a high prevalence of maternal HIV-infection.

## Supporting Information

S1 Table Risk factors for Group B streptococcus (GBS) invasive disease in HIV-infected and-uninfected mothers of GBS cases. (DOCX)

S2 Table Clinical and laboratory features of infants with invasive Group B streptococcal (GBS) disease. (DOCX)

S3 Table Baseline demographic characteristics of Group B streptococcus (GBS) cases and matched controls for 3 and 6 month visits. (DOCX)
