# Supplementary material for: Burden of Invasive Group B Streptococcus Disease and Early Neurological Sequelae in South African Infants
Source: PLoS One. 2015 Apr 7;10(4):e0123014. doi: 10.1371/journal.pone.0123014 (PMC4388823; doi:10.1371/journal.pone.0123014)
Supplement: S1 Table — (DOCX) [file pone.0123014.s001.docx]

S1 Table: Risk factors for Group B streptococcus (GBS) invasive disease in HIV-infected and –uninfected mothers of GBS cases

| Risk factors | HIV-infected, n=41 | HIV-uninfected, n=61 | OR (95%CI)^1^ | p-value |
| --- | --- | --- | --- | --- |
| Prematurity (<37 weeks) | 12 (29.3) | 21 (34.4) | 0.79 (0.30-2.00) | 0.585 |
| Prolonged ROM (>18hours)^2^ | 5/31 (16.1) | 11/53 (20.8) | 0.73 (0.18-2.63) | 0.775 |
| Maternal fever (≥38.0 °C) | 0/25 (0) | 1/52 (1.9) |  | 0.999 |
| Offensive liquor | 3/32 (9.4) | 9/58 (15.5) | 0.56 (0.09-2.52) | 0.527 |
| GBS Bacteriuria | 23/38 (60.5) | 22/51 (43.1) | 2.02 (0.79-5.20) | 0.105 |

^1^OR(95%CI)- calculated Odds ratio with 95% confidence using Fischer exact test comparing cases and controls, ^2^ Prolonged ROM(>18 hours)- prolonged rupture of membranes.
