# Supplementary material for: Burden of Invasive Group B Streptococcus Disease and Early Neurological Sequelae in South African Infants
Source: PLoS One. 2015 Apr 7;10(4):e0123014. doi: 10.1371/journal.pone.0123014 (PMC4388823; doi:10.1371/journal.pone.0123014)
Supplement: S2 Table — (DOCX) [file pone.0123014.s002.docx]

S2 Table: Clinical and laboratory features of infants with invasive Group B streptococcal (GBS) disease

|  | All cases,  n=122 | EOD^1^,  n=66 | LOD^2^,  n=56 | OR(95%CI)^3^ | p-value^4^ |
| --- | --- | --- | --- | --- | --- |
| Signs and symptoms |  |  |  |  |  |
| Respiratory distress | 75 (61.5) | 55 (83.3) | 20 (35.7) | 0.11 (0.04-0.28) | <0.001 |
| Poor feeding | 24 (19.7) | 2 (3.0) | 22 (39.3) | 20.71 (4.54-187.69) | <0.001 |
| Irritability | 33 (27.1) | 4 (6.1) | 29 (51.8) | 16.65 (5.03-69.74) | <0.001 |
| Lethargy | 23 (18.9) | 7 (10.6) | 16 (28.6) | 3.37 (1.17-10.51) | 0.012 |
| Apnea | 13 (10.7) | 6 (9.1) | 7 (12.5) | 1.43 (0.38-5.50) | 0.543 |
| Seizures | 13 (10.7) | 6 (9.1) | 7 (12.5) | 1.43 (0.38-5.50) | 0.543 |
| Increased tone | 21/119 (17.7) | 9/63 (14.3) | 12/56 (21.4) | 1.63 (0.57-4.82) | 0.308 |
| Decreased tone | 16/119 (13.5) | 9 /63 (14.3) | 7/56 (12.5) | 0.86 (0.25-2.82) | 0.776 |
| Temperature |  |  |  |  |  |
| Median(range) | 36.8 (33.2-40) | 36.6 (33.2-38) | 37.5 (35.8-40) |  | <0.001 |
| ≥38°C | 24 (19.7) | 2 (3.0) | 22 (39.3) | 20.71 (4.54-187.69) | <0.001 |
| ≤35.5°C | 5 (4.1) | 5 (7.6) | 0 (0) |  | 0.062 |
| Intensive/High care |  |  |  |  |  |
| Mechanical Ventilation | 19 (15.6) | 10 (15.2) | 9 (16.1) | 0.93 (0.31-2.84) | 0.889 |
| CPAP^5^ | 6 (4.9) | 6 (9.1) | 0 (0) |  | 0.031 |
| Inotropic support | 8 (6.6) | 5 (7.6) | 3 (5.4) | 0.69 (0.10-3.75) | 0.725 |
| Markers of infection |  |  |  |  |  |
| WCC^6^ | n=120 | n=64 | n=56 |  |  |
| Median(range)x10^9^/l | 11.8 (1.2-36.2) | 13.7 (2.4-36.2) | 8.0 (1.2-35.7) |  | 0.003 |
| WCC>20x10^9^/l | 27 (22.5) | 17 (26.6) | 10 (17.9) | 0.60 (0.22-1.57) | 0.255 |
| WCC<5x10^9^/l | 29 (24.2) | 8 (12.5) | 21 (37.5) | 4.20 (1.56-12.08) | 0.001 |
| CRP^7^ | n=105 | n=59 | n=46 |  |  |
| Median(range)mg/l | 31.0 (0-351.0) | 18.0 (0-277.0) | 43.5 (1.0-351.0) |  | 0.002 |
| CRP>10mg/l | 69 (65.7) | 35 (59.3) | 34 (73.9) | 1.94 (0.78-4.96) | 0.118 |
| CRP>40mg/l | 45 (42.9) | 18 (30.5) | 27 (58.7) | 3.24 (1.34-7.87) | 0.004 |

^1^EOD- Early-onset disease, ^2^LOD- Late-onset disease, ^3^OR(95%CI)- calculated odds ratio with 95% confidence comparing LOD to EOD, ^4^p-value- using Chi-squared, Fischer exact or Wilcoxon rank-sum (Mann-Whitney) test, ^5^CPAP- Continuous positive airway pressure, ^6^WCC- White cell count, ^7^CRP- C-reactive protein.
