# Supplementary material for: Burden of Invasive Group B Streptococcus Disease and Early Neurological Sequelae in South African Infants
Source: PLoS One. 2015 Apr 7;10(4):e0123014. doi: 10.1371/journal.pone.0123014 (PMC4388823; doi:10.1371/journal.pone.0123014)
Supplement: S3 Table — (DOCX) [file pone.0123014.s003.docx]

S3 Table: Baseline demographic characteristics of Group B streptococcus (GBS) cases and matched controls for 3 and 6 month visits

|  | **All** | | | **EOD^1^** | | | **LOD^2^** | | |
| --- | --- | --- | --- | --- | --- | --- | --- | --- | --- |
| **3 month visit** | **Cases, n=68** | **Controls, n=262** | **p-value** | **Cases, n=37** | **Controls, n=109** | **p-value** | **Cases, n=31** | **Controls ,n=153** | **p-value** |
| **Gestation** |  |  |  |  |  |  |  |  |  |
| ≥37 weeks | 48 (70.6) | 193 (73.7) | 0.611 | 24 (64.9) | 72 (66.1) | 0.895 | 24 (77.4) | 121 (79.1) | 0.836 |
| <37 weeks | 20 (29.4) | 69 (26.3) |  | 13 (35.1) | 37 (33.9) |  | 7 (22.6) | 32 (20.9) |  |
| Median(Range) | 39.5 (28.0-43.0) | 38.8 (28.0-44.0) | 0.525 | 38.4 (28.0-42.0) | 38.2 (30.0-44.0) | 0.959 | 40.0 (29.3-43.0) | 39.1 (28.0-44.0) | 0.134 |
| **Birth weight** |  |  |  |  |  |  |  |  |  |
| ≥2500 grams | 48 (70.6) | 204 (77.9) | 0.208 | 26 (70.3) | 73 (67.0) | 0.711 | 22 (71.0) | 131 (85.6) | 0.047 |
| <2500 grams | 20 (29.4) | 58 (22.1) |  | 11 (29.7) | 36 (33.0) |  | 9 (29.0) | 22 (14.4) |  |
| Median(Range) | 2903 (870-4155) | 3008 (1195-4315) | 0.066 | 2895 (870-4155) | 2870 (1195-3955) | 0.973 | 2915 (1415-3610) | 3105 (1465-4315) | 0.010 |
| **Gender** |  |  |  |  |  |  |  |  |  |
| Male | 41 (60.3) | 134 (51.2) | 0.178 | 20 (54.1) | 62 (56.9) | 0.765 | 21 (67.7) | 72 (47.1) | 0.036 |
| **HIV exposure** |  |  |  |  |  |  |  |  |  |
| HIV-exposed | 29 (42.6) | 122 (46.6) | 0.563 | 10 (27.0) | 30 (27.5) | 0.953 | 19 (61.3) | 92 (60.1) | 0.904 |
| HIV-unexposed | 39 (57.4) | 140 (53.4) |  | 27 (73.0) | 79 (72.5) |  | 12 (38.7) | 61 (39.9) |  |
| **Clinical presentations** |  |  |  |  |  |  |  |  |  |
| GBS meningitis | 19 (27.9) |  |  | 2 (5.4) |  |  | 17 (54.8) |  |  |
| Perinatal asphyxia | 9 (13.2) |  |  | 9 (24.3) |  |  |  |  |  |
| Ventilated | 3 (4.4) |  |  | 2 (5.4) |  |  | 1 (3.2) |  |  |
|  |  |  |  |  |  |  |  |  |  |
| **6 month visit** | **Cases, n=68** | **Controls, n=232** | **p-value** | **Cases, n=36** | **Controls, n=96** | **p-value** | **Cases, n=32** | **Controls ,n=136** | **p-value** |
| **Gestation** |  |  |  |  |  |  |  |  |  |
| ≥37 weeks | 50 (73.5) | 185 (79.7) | 0.274 | 24 (66.7) | 71 (74.0) | 0.406 | 26 (81.3) | 114 (83.8) | 0.725 |
| <37 weeks | 18 (26.5) | 47 (20.3) |  | 12 (33.3) | 25 (26.0) |  | 6 (18.7) | 22 (16.2) |  |
| Median(Range) | 40.0 (28.0-43.0) | 39.0 (26.2-44.0) | 0.588 | 38.2 (28.0-42.0) | 38.4 (30.0-44.0) | 0.446 | 40.0 (28.0-43.0) | 39.2 (26.2-44.0) | 0.042 |
| **Birth weight** |  |  |  |  |  |  |  |  |  |
| ≥2500 grams | 51 (75.0) | 189 (81.5) | 0.241 | 26 (72.2) | 71 (74.0) | 0.841 | 25 (78.1) | 118 (86.8) | 0.217 |
| <2500 grams | 17 (25.0) | 43 (18.5) |  | 10 (27.8) | 25 (26.0) |  | 7 (21.9) | 18 (13.2) |  |
| Median(Range) | 2920 (870-4155) | 3039 (1170-3955) | 0.072 | 2903 (870-4155) | 2920 (1405-3955) | 0.690 | 2938 (1200-3610) | 3093 (1170-395) | 0.039 |
| **Gender** |  |  |  |  |  |  |  |  |  |
| Male | 43 (63.2) | 124 (53.5) | 0.153 | 21 (58.3) | 55 (57.3) | 0.914 | 22 (68.8) | 69 (50.7) | 0.066 |
| **HIV exposure** |  |  |  |  |  |  |  |  |  |
| HIV-exposed | 27 (39.7) | 100 (43.1) | 0.618 | 9 (25.0) | 24 (25.0) | 0.999 | 18 (56.3) | 76 (55.9) | 0.970 |
| HIV-unexposed | 41 (60.3) | 132 (56.9) |  | 27 (75.0) | 72 (75.0) |  | 14 (43.7) | 60 (44.1) |  |
| **Clinical presentations** |  |  |  |  |  |  |  |  |  |
| GBS meningitis | 17 (25.0) |  |  | 2 (5.6) |  |  | 15 (46.9) |  |  |
| Perinatal asphyxia | 9 (13.2) |  |  | 9 (25.0) |  |  | - |  |  |
| Ventilated | 4 (5.9) |  |  | 2 (5.6) |  |  | 2 (6.3) |  |  |

^1^EOD- Early-onset disease, ^2^LOD- Late-onset disease.
